# Students’ self-reported well-being under corona measures, lessons for the future

**DOI:** 10.1016/j.heliyon.2022.e08733

**Published:** 2022-01-11

**Authors:** Ralph Meulenbroeks, Wouter R. van Joolingen

**Affiliations:** Freudenthal Institute, Utrecht University, the Netherlands

**Keywords:** Online education, Basic psychological needs, Student well-being, COVID-19, Student support

## Abstract

As education was forced to go fully online in early 2020 as a consequence of the imposed lockdowns, concerns were raised related to student well-being. This study examines student well-being at the science faculty of a large urban university in the Netherlands within the framework of Basic Psychological Need Theory, a sub-theory of Self-Determination Theory. A mixed-methods approach was adopted, combining the results of an online student survey with 16 Likert-scale questions as well as two open ended questions (2228 participants, corresponding to a response rate of 32%) with a student focus group interview. The Likert-scale questions were subjected to factor analyses and reveal problems with well-being in four areas: study, personal worries, personal well-being, and societal worries. The analyses of the answers to the open ended questions as well as the focus group data show that students are positive on the autonomy offered by the inherent flexibility of online education, e.g., as a consequence of reduced travel time. However, the psychological needs of competence and relatedness are seriously undermined during times of lockdown, mainly because of poorer student-teacher interaction, lack of structure, loneliness, and helplessness. Students state that they need more support in these areas, for example by on-campus meetings whenever possible, psychological support, improvements in online education, online social events, adequate communication, and leniency in the interpretation of regulations and deadlines. Implications are discussed.

## 1 Introduction

In early 2020, education at all levels and across the globe was forced to make a very sudden transition. Although the restrictions on social interaction differed per country and region, education as the world had known it was forced to ‘come to a screeching halt’ (Ellis et al., 2020). In order to keep some kind of continuity most educational institutions worldwide opted for fully online education. This decision came with important and often pressing consequences for achievement, equity, teaching methods, management, and especially well-being of teachers, management, parents with children in school, and -of course-students (Ellaway et al., 2020; Hoyt et al., 2021; Meulenbroeks et al., 2021; Williamson et al., 2020).

A review on the effects of quarantine demonstrates that the psychological effects in terms of confusion, anger, and post-traumatic stress can intense and long-lasting (Brooks et al., 2020). Furthermore, a longitudinal study in China shows that even without being quarantined in a strict sense, the well-being of the general population suffered under the imposed and enforced social distancing. Common reactions such as depression, anxiety, and self-reported stress were reported by about one-half of the respondents in this study (Wang et al., 2020). Importantly, these symptoms were not observed to decline over a time period of one month.

Among the subjects of the present study, students in higher education, studies confirm a deterioration of well-being since the onset of the COVID-19 measures in early 2020, with reports of as much as 68% of higher education students experiencing such a deterioration. Concerns include worries about returning to normal and students’ graduation process (Arslan and Allen, 2021; Lyons et al., 2020). Some students were able to cope better than others. A strong sense of meaning in life and psychological flexibility reportedly helped these (undergraduate) students in dealing with COVID-related stress (Genç and Arslan, 2021).

At a large university in the center of the Netherlands, the context of this study, restrictions for higher education started on March 16^th^, 2020, with all on-campus activities being suspended effectively until September (Rijksoverheid, 2021). In the wake of the transition, several surveys and case studies were conducted on the effects of the sudden transition on students (e.g., Bakker and Wagner, 2020; Meulenbroeks, 2020), bringing to light difficulties in line with the studies mentioned so far. At the beginning of the academic year 2020–21. small-scale on-campus activities were allowed within severe restrictions such as the wearing of face masks and maintaining a 1.5 m distance between persons. Large meetings were not allowed. For many first-year students this implied that they had to start their fresh study without ever physically visiting their university. As the second wave of infections hit the Netherlands in October 2020, all on-campus activities were again suspended until the end of April 2021. At the time this article is written (July 2021), on-campus activities are again possible on a small scale.

At the science faculty of the university, student advisors in particular were interested in the way the science students were handling education, especially with a large number of newly arrived undergraduate students that had never enjoyed ‘regular’ on-campus education. Therefore, in November 2020, during the second lockdown a large survey was conducted among all 6950 graduate and undergraduate students of the science faculty. Of this sample, 2228 students responded to this online survey, answering open and closed questions in an online questionnaire. The response rate of 32%, satisfactory and typical for an online questionnaire (Nulty, 2008), prompted the authors of this article to closely analyze the results of the survey and follow up on it with a focus group of volunteer students. This study therefore employs a mixed-methods approach.

The theoretical lens chosen for this approach is self-determination theory, more explicitly basic psychological need theory (Ryan and Deci, 2017; Vansteenkiste et al., 2020). This mini-theory within the framework of self-determination theory is devoted to the study of how well-being is related to the support or thwarting of three basic psychological needs: autonomy, competence, and relatedness.

The research question is: *What do undergraduate and graduate science students report on their well-being and psychological need support during predominantly online education?*

## 2 Theoretical framework

Well-being is a concept with many dimensions. In the hedonic view it is defined mainly as the absence of negative affect, combined with the presence of positive affect. Generally this is related to what people report on their feelings of either happiness or being fulfilled (Diener, 2000; Kahneman and Krueger, 2006).

An influential alternative approach to the concept of well-being goes back to the days of Aristotle. In this eudaimonic vision, seeking pure happiness, satisfaction, and pleasure is not considered to be the primary road to well-being (Ryan and Martela, 2016; Waterman, 1990). Instead, eudaimonia suggests that person's activities be directed towards aims that are inherently valuable (Huppert and So, 2013), goals that are, indeed, worthy as they serve something that is bigger than the person. Very often, these goals are related to societal issues and self-actualization (Maslow and Rogers, 1979). This more comprehensive, eudaimonic form of well-being is sometimes referred to as flourishing. In this vision, happiness does not equal well-being, it is a symptom of well-being (Ryan and Deci, 2017).

Within self-determination theory (SDT) this state of eudaimonic well-being is intimately linked to support of three universal, innate, psychological needs. The underlying assumption is that support of these needs (autonomy, competence, and relatedness) fosters growth and makes humans flourish. In real life, people are confronted with huge differences in biological or genetic inclinations, social and economic background, and political systems in which they are living and working. The support of basic psychological needs, however, has been shown to be a universal mediating factor if these circumstances are less than favorable (DeHaan et al., 2016; Legate et al., 2015). The influence of basic psychological need support and well-being is described in one of SDT's mini-theories: Basic Psychological Need Theory or BPNT (Ryan and Deci, 2017; Vansteenkiste et al., 2020).

BPNT states that each human being, having fulfilled the basic *physical* needs of food, shelter, and sex, has three basic *psychological* needs. The fact that these psychological needs are innate and universal has important implications for well-being. When any one of these needs is not supported, well-being (and motivation) in any individual, independent of context or demographic situation, will suffer. Well-being, or flourishing in the eudaimonic view, requires that all three needs are supported.

We will now examine definitions of these psychological needs and attempt to translate them to the case of university students in lockdown.

*Autonomy*… ‘refers to the experience of volition and willingness. When satisfied, one experiences a sense of integrity as when one's actions, thoughts, and feelings are self-endorsed and authentic. When frustrated, one experiences a sense of pressure and often conflict, such as feeling pushed in an unwanted direction.’ (Vansteenkiste et al., 2020, p.3).

The bare fact that the lockdown is put into place by the government and interpreted by the university clearly thwarts the need for autonomy: the student -as well as the general population-has no volition in this process and sees the possibilities for free behavior dwindle. However, fully online education also has its benefits in terms of student autonomy: with its reliance on videos and online work, it can be accessed at any time. Travel to and from a specific building is not necessary anymore. The student can, for example, choose to follow and do the online work in bed, at another house, in nature, etc.

*Competence*… ‘concerns the experience of effectiveness and mastery. It becomes satisfied as one capably engages in activities and experiences opportunities for using and extending skills and expertise. When frustrated, one experiences a sense of ineffectiveness or even failure and helplessness.’ (Vansteenkiste et al., 2020, p.3).

In education, the support of competence is crucial (Deci et al., 2001), since the understanding, digestion, and application of content is central to the process. Capably engaging in educational activities requires support, or scaffolding, by the teacher (Quintana et al., 2004) and, often, working together with other students, e.g., in inquiry-based learning (Capps and Crawford, 2013). In fully online education it can be expected that support in terms of asking questions and having them answered quickly, suffers (Meulenbroeks, 2020; Shu and Gu, 2018). On the other hand, online formats such as recorded lectures can also be seen as advantageous from this point, since they offer the possibility to scroll forwards and backwards in order to skip passages that are already known and to revisit parts that are considered difficult.

*Relatedness*… ‘denotes the experience of warmth, bonding, and care, and is satisfied by connecting to and feeling significant to others. Relatedness frustration can come with a sense of social alienation, exclusion, and loneliness.’ (Vansteenkiste et al., 2020, p3, p3).

Even though the advent of social networks has enabled almost instantaneous and global communication, relatedness is by no means automatically supported in online environments and social networks (Chen et al., 2021). The effects of lockdown on relatedness are expected to be mainly negative since social contact is basically limited to online interaction and interaction with family or, in the case of students, a group of close friends or housemates. Indeed, as was mentioned in the introduction, the effects of lockdown on the general population, e.g., loneliness and depression (Wang et al., 2020), appear to mirror thwarting of the psychological need for relatedness.

On the basis of BPNT we can formulate our hypothesis:

During primarily online education, students will experience a diminished sense of well-being, as their universal psychological needs are thwarted by imposed measures in order to contain the spread of the COVID-19 virus, a lack of direct interaction during education, and severe restrictions on social contact. These negative effects are mediated by a heightened sense of autonomy in terms of place and time, advantages of online education in terms of competence support, and closer relations with a small group of intimate friends or relatives.

On top of this, students may feel their physical needs threatened by a pandemic that may directly affect their health and the health of their relatives or friends.

## 3 Method

### 3.1 Context

This mixed-methods study was conducted at the science faculty of a large urban university in the Netherlands. The science faculty encompasses six departments: mathematics, physics, chemistry, biology, information and computing sciences, and pharmaceutical sciences. It features a total of about 6950 undergraduate and graduate students. The survey did not collect demographic data such as gender, age, or nationality, but students were asked to indicate whether they were master of bachelor students. Of the total number of students 40 % were master students. Among the 2228 respondents we find 1305 bachelor and 905 master students, resulting in a very similar percentage of 41 % master students.

### 3.2 Data collection

Within this mixed-methods study, the data collection process consisted of three parts:

#### 3.2.1 Online questionnaire

A 16-item 5-point Likert scale online questionnaire on student well-being was designed by the faculty's chief student advisor and administered in November 2020, during the second lockdown and suspension of all physical meetings at the university. It was sent to all 6950 graduate and undergraduate students of the science faculty and resulted in a response rate of 32% (2228 participants). The full questionnaire can be found in Table 4. The root question was on how students perceived their own well-being under the conditions imposed by the measures due to the corona pandemic. Well-being was operationalized using questions such as: ‘Because of corona measures I feel more relaxed’ and ‘Because of corona measures I feel more lonely than before.’ Note that this questionnaire in itself was not based on SDT or BPNT. It was mainly the satisfactory response rate that prompted the authors to analyze the results with an SDT-lens and extend it with qualitative data.

#### 3.2.2 Open ended questions in the questionnaire

The same online questionnaire featured two open ended questions, aimed at obtaining information on possible positive (side) effects of the lockdown and possible solution directions:

a. Which advantages of the corona measures do you see?
b. What would you like your faculty to do in order to improve student well-being in corona times?

Students could choose to either answer or ignore these questions as they were not a mandatory part of the questionnaire.

#### 3.2.3 Focus group interview

After initial data analyses (see below), science faculty students were randomly and voluntarily selected for a focus group interview. This qualitative part of the mixed-methods approach was adopted (Almeida et al., 2017; Morgan, 1996) since the research question requires in-depth information on the various dimensions of the issue, alongside quantitative information. Since the answers to the open ended questions in the large questionnaire had already been analyzed at this point, a relatively open, qualitative method was judged to be most likely to accommodate responses in areas that needed elucidation.

Six students volunteered to participate in the focus group. However, due to COVID-related issues only three students eventually showed up in the online environment chosen for the interview at the designated time in May 2021. The decision was made to carry on with the focus group with this limited number of students, a limited generalizability notwithstanding. Even though this low number of participants restricted the level of interaction, it did still allow for an in-depth discussion between the three participants. The participants happened to represent different categories: two undergraduate students: physics (M) and biology (F), and one international graduate student in environmental biology (F). The focus group was conducted in English and had a duration of 50 min.

The guiding questions for the focus group were explicitly based on BPNT, but worded in a general way, e.g.:

1. Has this period of mainly online education made you feel more -or less-in control of your life and study?
2. Has this period of online education made it easier of harder for you to pursue your study?
3. To what extent has this period of mainly online education influenced your relations, both social and academic?

The full interview protocol, including the informed consent statement, is given in Figure 6 at the end of this publication. During the interview, conducted by one of the authors, the students were left to interact freely, the researcher only intervened in order to ask the guiding questions and some follow-up questions such as: ‘Could you explain, why?’ or ‘Could you give an example?‘.

### 3.3 Data analysis

#### 3.3.1 Quantitative analyses

In the quantitative analysis we only used the 2150 (out of 2228) responses for which all sixteen Likert-scale items were answered. For these items we computed means and standard deviations (treating them as interval data) to get an overall impression of students' self-reported well-being. In order to detect dimensions in students’ reported well-being, using the Likert-type items, we performed a principal component analysis on these sixteen items, with oblimin rotation.

#### 3.3.2 Open ended questions in the questionnaire

Out of 2228 respondents, 1975 entered answers to at least one of the two open ended questions of the questionnaire (88.6 % of the respondents). Some answers were as short as a few words (e.g., ‘less travel’), whereas others had 100 words or more, often addressing more than one issue. Open coding (bottom-up) was performed on the reponses, the longer answers often being assigned more than one code. A total of 2525 codes were found on the basis of the answers to question a (perceived advantages), whereas 1837 codes were found on the basis of the answers to question b (solution directions).

Second coding was performed on 100 randomly selected quotes for each question. This resulted in Cohen's kappa = 0.93 for the codes relating to question a and Cohen's kappa = 0.98 for question b, indicating near perfect intercoder agreement for both cases (Landis and Koch, 1977).

The answers to question a resulted in 11 bottom-up categories, whereas the answers to question b generated 10 categories. The categories for question a are given in Table 1, those for question b in Table 2. The basic psychological need associated with each (bottom-up) code by the authors, has been given in the last column of the tables.

**Table 1:** Categories identified by bottom-up coding the answers to the open question a: Which advantages of the corona measures do you see? BPN refers to the Basic Psychological Need being associated with the category, as judged by the authors.

Category	Description	Typical quote	BPN
TRV	Less time spent travelling	Because of online lectures, I save about 9 h of travel time a week, time that I can use for my study.	A
COV	Limit the spread of COVID-19	Less infections, less deaths	PHYS
ADV	Specific advantages of online education, e.g., lectures being recorded	Is it nice that the lectures are being recorded, so you can pause them during playback, e.g., to think about what has just been explained.	C
FLX	Flexibility	Manage your own time	A
TIM	More time available in general	It gives you time to do the things you normally would have little or no time to do.	A
NON	No advantages at all	Nothing. This is a period to get through, not stay in.	N/A
HLT	Healthier lifestyle, including a better day/night rhythm	More sleep (because to be on time for a 0900 lecture I needed to get up at 0745, now it is 0845 and because cafes are closed I go to bed earlier).	PHYS
MSO	More social activities with specific people, less social pressure	Less social pressure (fear of missing out), more time for a smaller group of people who really matter.	R
DIS	Better concentration and less distraction	There are a lot less social distractions anyhow.	C
AWR	Awareness of the things that truly matter	One advantage that I hope will come out of this, is more awareness about the way we deal with nature and biodiversity. For example, there is a large decline in CO2 production and hopefully in the future we will be more aware of the fact that we need to need to be less disruptive with nature and put less of a burden on it.	R
LOE	Learning about setting up online education	I think the corona measures put more emphasis on the digitization of education and work. As we are being confronted with this much more strongly now, it becomes clear what can be improved and criticisms are taken into account. I haven't really experienced that before.	C

A = autonomy; C = competence; R = relatedness. PHYS refers to physical needs.

**Table 2:** The categories identified by bottom-up coding of the answers to the open ended question b: What would you like your institute to do in order to improve student well-being in corona times? BPN refers to the basic psychological need being associated with the category, according to the authors.

Category	Description	Typical quote	BPN
OFF	Return to physical meetings asap	More offline education or more contact hours. Especially last semester I missed the contact with fellow students and the teacher in a course.	R
IOE	Improve the quality of online education by supporting teachers	Better digital education, or ways in which we can work together at a distance.	C
LEN	Leniency with regulations, e.g. missed deadlines	More possibilities for extending deadlines and doing resits of exams.	C
SUP	More support for students in organizing online education	Offer online support as much as possible.	C
OSA	Organize online social activities	Organize something to get to know your fellow students in a course.	R
PSY	Offer psychological support	Pay more attention for people who are maybe not feeling well. Actively offer psychological support.	R
COM	Better and more frequent communication	In communication emails, give a short summary of the main new developments in order to give me a quick overview. I can read the reasoning later.	C
OKA	It is fine as it is	Nothing. I am fine.	N/A
NOS	No suggestions at all	I have no idea what they can do.	N/A
FIN	Financial compensation for lack of physical meetings	Financial compensation whenever possible.	A

A = autonomy; C = competence; R = relatedness.

The categories identified for the answers to question b are given in Table 2.

#### 3.3.3 Focus group interview

The focus group interview was videotaped and transcribed verbatim. The full text was then read several times and relevant, self-contained quotes were selected. A total of 29 self-contained codes was found. Since the focus group was primarily intended to add ‘color’ to the answers to the open ended questions in the questionnaire and the bottom-up coding of the answers to these open ended questions suggested strong links with BPNT (see Tables 1 and 2), the quotes from the focus group interview were classified following a top-down coding protocol based on the theoretical framework of BPNT. Six categories were used, corresponding to the three psychological needs being either thwarted or supported. All codes were subjected to second coding, resulting in a Cohen's kappa of 0.87, again indicating near-perfect interrater agreement. Table 3 gives the categories along with their description and a typical quote.

**Table 3:** The categories used for coding the focus group results. The category descriptions are taken from (Vansteenkiste et al., 2020). Pluses or minuses refer to supporting or thwarting the corresponding psychological need.

Category	Description	Typical Quote
Competence +	Competence ​concerns the experience of effectiveness and mastery. It becomes satisfied as one capably engages in activities and experiences opportunities for using and extending skills and expertise.	Actually I do feel competent [in pursuing my study], I succeeded in my courses even better than before the lockdown
Competence -	When frustrated, one experiences a sense of ineffectiveness or even failure and helplessness.	So it started this loop like: I am so behind with work, I don't want to do anything and then I didn't do anything and I felt like a loser.
Autonomy +	Autonomy ​refers to the experience of volition and willingness. When satisfied, one experiences a sense of integrity as when one's actions, thoughts, and feelings are self-endorsed and authentic.	I had more freedom to schedule my own time [and] my days.
Autonomy -	When frustrated, one experiences a sense of pressure and often conflict, such as feeling pushed in an unwanted direction.	I had to transform all that work to something I could do remotely, which was mostly like computer work and statistics, which is something I really don't like.
Relatedness +	Relatedness ​denotes the experience of warmth, bonding, and care, and is satisfied by connecting to and feeling significant to others.	I am really happy in my student home, with 10 people, like five guys and five girls and I really get a lot of support out of that. Everyone is quite optimistic and doing their own thing here. I get a lot of comfort out of that, and stability.
Relatedness -	Relatedness frustration can come with a sense of social alienation, exclusion, and ​loneliness.	We're still in lockdown, many of my friends are gone […]. I felt basically alone. Also, the international students, most of them were back home […].

## 4 Results

### 4.1 Quantitative analysis

Table 4 displays the mean answers to the Likert-scale questions on student well-being, sorted in descending order. One outstanding result is the high value for less social interactions, which is very near the maximum score. Other high scoring questions relate to studying online (concentration and structuring) and worries, mainly for family and perspective on the study. On the lower of the scale, students disagree with a positive influence of the corona measures on well-being and being relaxed. Less worry appears to exist on the financial situation and personal health.

**Table 4:** Mean scores of Likert-scale items on student well-being, ordered from highest to lowest.

Question	Mean	SD
Because of corona measures I have less social interactions	4.52	0.76
Because of corona measures I have trouble to concentrate	3.57	1.22
Because of corona measures I have trouble structuring my day	3.54	1.27
Because of corona measures I am more lonely than before	3.53	1.11
Because of the corona virus I worry about the health of family and friends	3.49	1.16
I worry that the current situation limits the options I have within my educational program	3.49	1.24
Because of the corona virus I worry more than before	3.32	1.19
I have trouble following online education	3.24	1.26
I worry that the current situation will influence my career	3.06	1.31
I worry that the current situation will influence my graduation date	3.05	1.39
Because of corona measures I suffer from more depressive complaints than before	3.04	1.28
Because of corona measures I spend more time on my studies	2.72	1.15
I worry about my financial situation	2.57	1.34
I am worried about my health	2.53	1.20
Because of corona measures I am more relaxed	2.29	1.08
Corona measures positively influence my well-being	2.14	1.00

Note: mean values of 5 point likert scale, minimum = 1, maximum = 5. N = 2150.

The sixteen Likert-scale questions were analyzed using a Principal Component Analysis. After inspecting the scree plot and oblimin rotation, four factors, together explaining 57% of the variance, were identified. Table 5 displays the resulting pattern matrix. Based on the items that load to the factors we identified them as *problems with studying*, *personal worries*, *personal well-being*, and *societal worries*, expressing the main impacts that corona has on overall student well-being. A reliability analysis of the resulting scales showed Cronbach's Alphas of 0.776. 0.657, 0.742 and 0.743 for these scales respectively.

**Table 5:** Pattern matrix for the factor analysis on the sixteen Likert-scale questions. Shown are factor loadings with an absolute value greater than 0.25

Pattern Matrix				
	Component			
	problems with studying	personal worries	personal well-being	societal worries
Because of corona measures I spend more time on my studies	-0.773			
Because of corona measures I have trouble to concentrate	0.759			
Because of corona measures I have trouble structuring my day	0.758			
I have trouble following online education	0.659			
I am worried about my health		0.843		
Because of the corona virus I worry about the health of family and friends		0.775		
Because of the corona virus I worry more than before		0.554	-0.273	
Because of corona measures I am more lonely than before			0.788	
Because of corona measures I have less social interactions			0.752	
Corona measures positively influence my well-being	0.258		-0.567	
Because of corona measures I am more relaxed	0.251		-0.538	
Because of corona measures I suffer from more depressive complaints than before			0.529	
I worry that the current situation will influence my career				0.843
I worry that the current situation limits the options I have within my educational program				0.795
I worry that the current situation will influence my graduation date				0.760
I worry about my financial situation				0.591
*Extraction Method: Principal Component Analysis.*				
*Rotation Method: Oblimin with Kaiser Normalization.*				
*Rotation converged in 11 iterations.*				

### 4.2 Open ended questions in the online questionnaire

**Figure 1:**
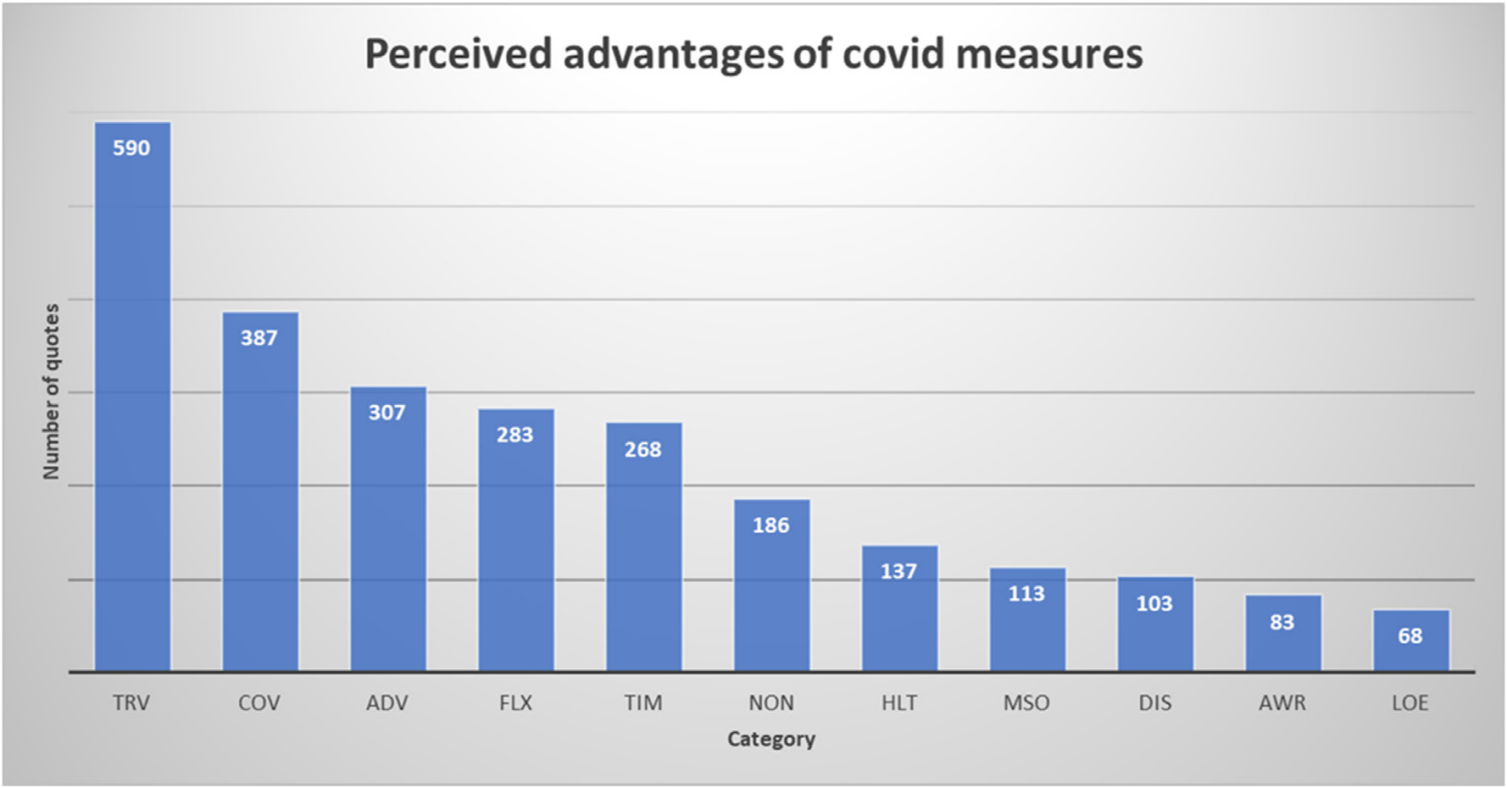
Perceived advantages of the COVID-measures as reported in the online questionnaire. The labels of the categories refer to Table 1.

Figure 1 displays the number of quotes per category in students answers to open ended question a: Which advantages of the corona measures do you see?

Three categories account for over half the number of quotes: reduced travel time (23.4%), prevention of infections (15.3%), and advantages of online education (12.2%). While the first two are self-explanatory and usually are expressed in short quotes (‘less travel time’ or ‘less infections’), students use more words to explain the advantages of online education:

*‘The lectures are online now, this offers the possibility of rewinding them and pause them if necessary.’*

The quotes within this category almost exclusively concern this feature of recorded lectures. Actually, no quotes explicitly address other advantages of online education. The aspects of flexibility (11.2%) and more time in general (10.6%) are often mentioned:

*‘Flexibility as to where and when I follow lectures and work on assignments.’*

*‘Having more free time to spend on hobbies, explore new fields, etc.’*

An important minority of 7.4% of the respondents explicitly indicate that they cannot come up with one single advantage of the situation.

The remaining five categories together account for 21.9% of the total number of quotes. They thus refer to perceived advantages that are less often mentioned, e.g., health issues (mostly a better day/night rhythm), less social distractions, and learning about online education in general. A small percentage of quotes (4.5%) refers to improvements in social interactions:

*‘I notice that I remain in contact with friends and family more consciously, I make (video) calls and check in [with them] more often.’*

and a sense of increased awareness of what really matters in life in general (3.3%):

*‘Maybe more contemplation about what makes you happy and what not.’*

**Figure 2:**
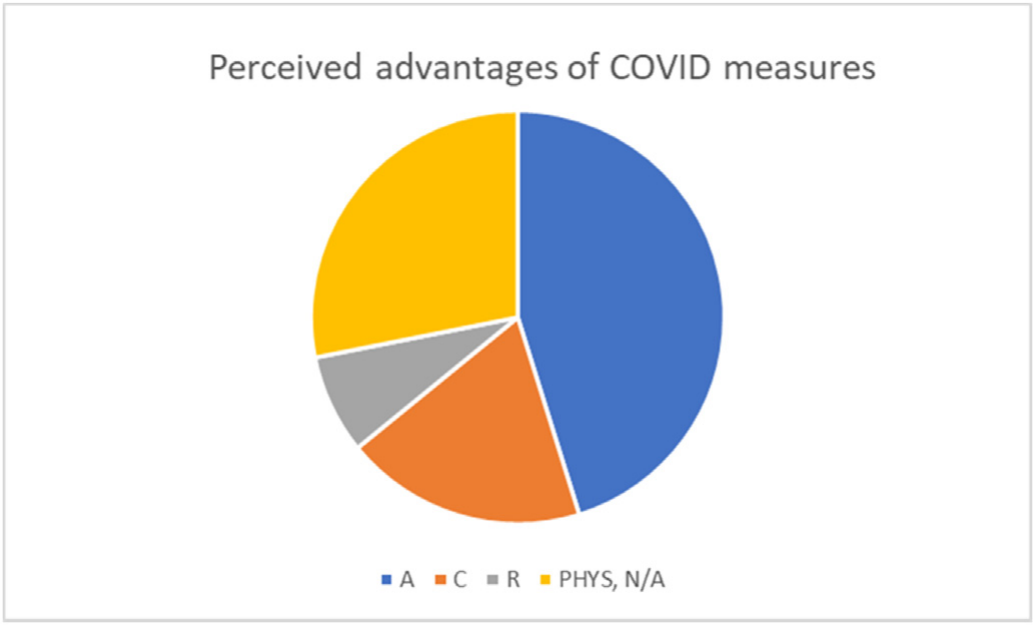
The distribution of the answers to question a over the different psychological needs in BPNT. A = autonomy; C = competence; R = relatedness.

If we apply the theoretical lens of BPNT (last column of Table 1) to these quotes, Figure 2 arises. It shows that the majority of quotes on perceived advantages can be related to the basic psychological need of autonomy (45.2%), with relatedness only accounting for 7.8% of these positive quotes.

**Figure 3:**
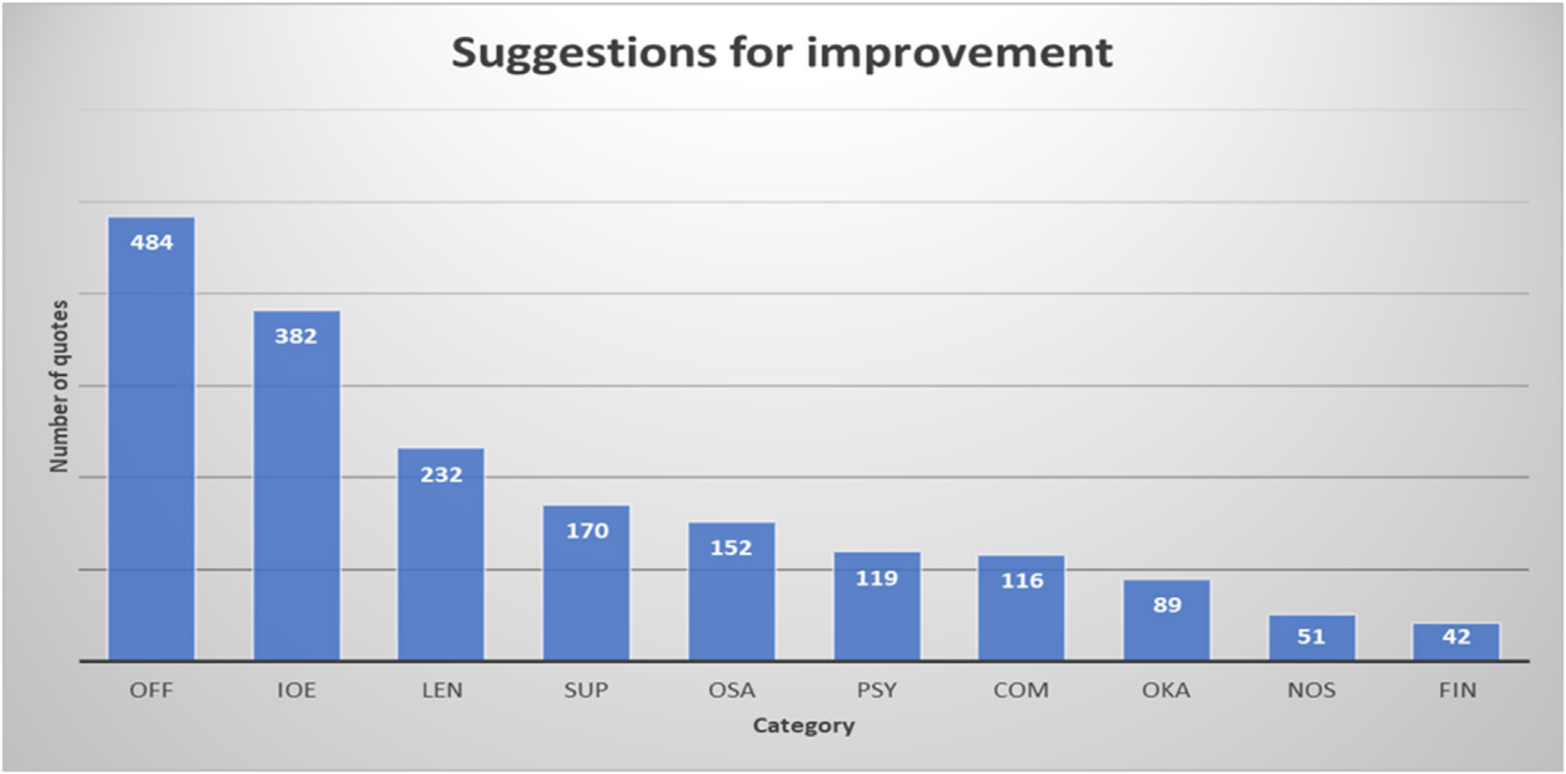
Suggestions for improving student well-being during periods of online education and lockdown as given by students in the online questionnaire. The labels of the categories refer to Table 2.

Figure 3 gives the number of quotes per category in students answers to open ended question b: What would you like your faculty to do in order to improve student well-being in corona times?

Also in the case of question b, three categories account for more than half of the quotes: return to campus (offline) education as soon as possible (26.3%), improving online education (20.8%) and leniency with regulations (12.6%, mainly relating to extending deadlines and decreasing high-stakes testing). Students’ have diverse suggestions on how to improve online education. For example, they promote support for teachers and ask for more diverse activities:

*‘Teach teachers how to use digital environments. This would decrease frustration for all parties concerned.’*

*‘Offer more diverse online education.’*

*‘I would propose more and more interesting alternatives in education. I am thinking of more online symposia/lectures on corona/study-related subjects.’*

Students express a preference for synchronous activities:

*‘[…] give actual lectures, not pre-recorded clips with subsequent quizzes.’*

*‘More interaction between teacher and student, e.g., during online working groups.’*

Students also recommend two types of support. First, support on how to organize their own online work and schedules (9.3% of quotes):

*‘Tips and tricks to study better at home, take into account that concentration is not always optimal.’*

Second, psychological support (6.5%):

*‘Extra courses about dealing with stress, more courses on how to relax. Personally I have had trouble with panic attacks that have increased during corona due to reduced social contacts.’*

*‘More personal attention, ask student show they are doing more often (1 on 1 conversation).’*

In line with this latter type of support, students advise implementation of online social activities (8.3%):

*‘For my study it would maybe be an idea to plan an [online] informal afternoon/evening with a nice pub quiz for the students to get to know each other a bit better.’*

A number of quotes (6.3 %) concerns the style and quality of communication by the faculty. Students recommend clear, concise, and frequent communication with the interpretation of the latest regulations:

*‘…be extra clear about what corona means for education and the students.’*

Only about one out of twenty quotes (4.8%) indicates that all is fine in the online education situation. An even smaller number (2.8%) offers no suggestions at all. The smallest percentage of quotes concerns financial compensation for the lack of on-campus education (2.3%).

**Figure 4:**
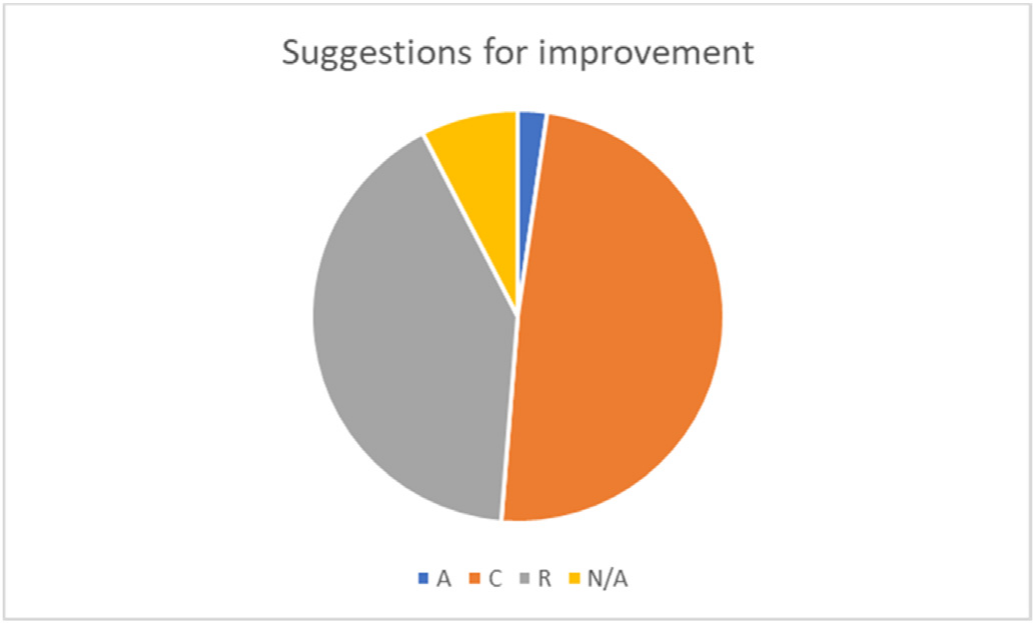
The distribution of the suggestions for improvement over the different psychological needs in BPNT. A = autonomy; C = competence; R = relatedness.

The answers to question b can also be categorized using the BPNT-lens (last column in Table 2). This results in Figure 4, which shows a very different picture from Figure 2. In the suggestions students almost exclusively refer to measures related to competence (49%) and relatedness (41.1%).

### 4.3 Focus group interview

**Figure 5:**
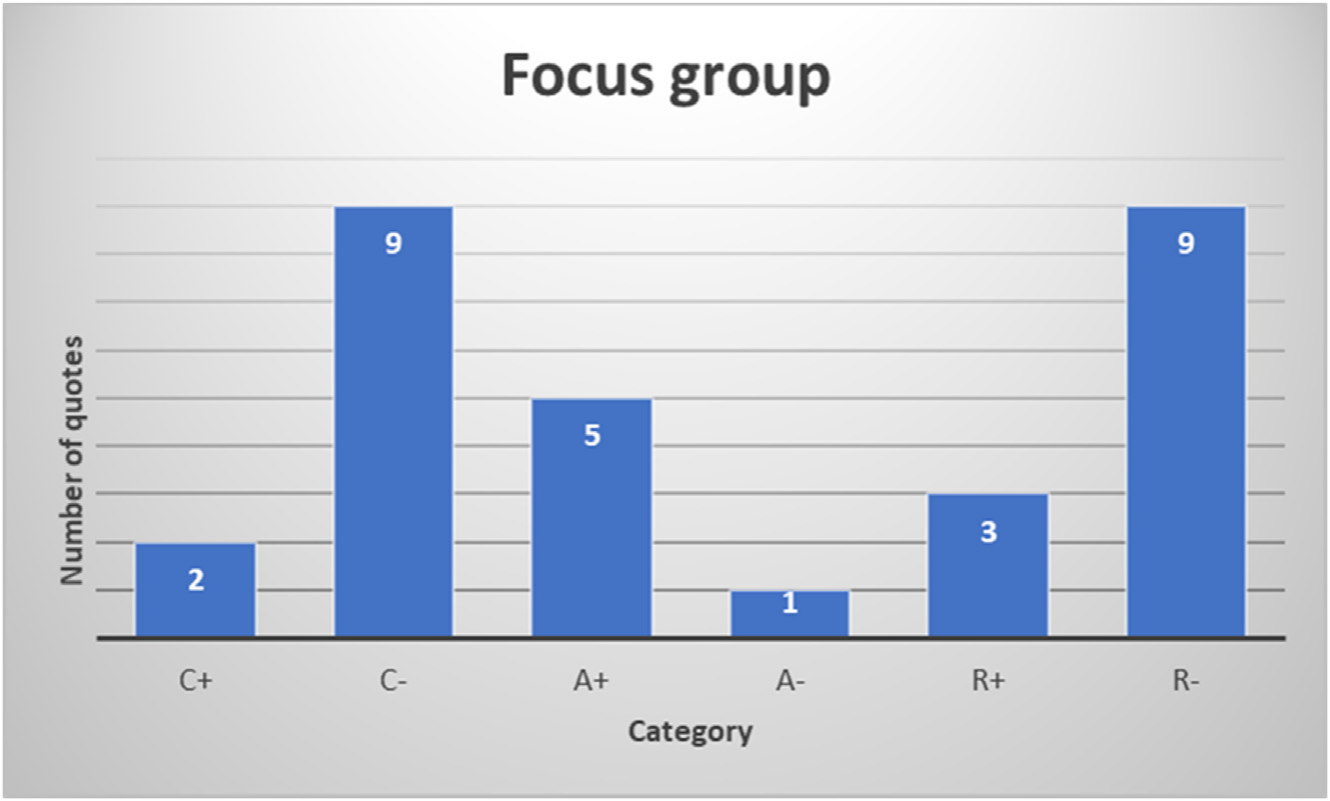
The results of the focus group interview. The categories are related to a psychological need (C: competence; A: autonomy; R: relatedness) being either supported (+) or thwarted (-).

Figure 5 shows the distribution of the 29 quotes from the focus group over the six categories from BPNT. The figure reflects similar results as the results of the open ended questions in the questionnaire: the majority of quotes (18) refers to thwarting of competence (9 quotes):

*‘My life is more structured when I feel I have to get up and prepare myself. [...] In the corona period, I am just at home the whole day. I don't have anything else to do [...]. So I felt that I just had this time and of course also the University was like: “oh, schedule your day, blah, blah, blah", but in the end I would just find myself like: "I just have the whole day", and I don't have anywhere to go and I don't have anything today except what I have to do so I would just get into procrastination.’*

and thwarting of the need for relatedness (9 quotes):

*‘The people you would hang out with that weren't really your friends but you would meet up [with-]in a group of friends. That kind of thing you cannot do online. [...] That kind of connection and relationship with people is, I think, lost. Having lost that over the past year I think is quite heavy because it is the small connections that make quite a difference in the general well-being.’*

The positive comments on relatedness are dealing with the quality of interaction with close friends and relatives:

*‘I am really happy in my student home, with 10 people, like 5 guys and 5 girls and I really get a lot of support out of that. Everyone is quite optimistic and doing their own thing here. I get a lot of comfort out of that, and stability.’*

This sentiment was also encountered in some of the answers to open ended question a.

Autonomy is often (in this case, explicitly so) perceived as being supported in online education (5 quotes):

*‘I have more time to do my own thing. I think that actually gives me a sense of [...] autonomy because I am quite an evening person. I really like the feeling of being productive in the morning but I don't like to wake up early in the morning [and] I have a hard time getting into that structure. That, I guess, gives me more sense of control over my life.’*

Examples of quotes on support for competence and thwarting of autonomy can be found in Table 3.

## 5 Conclusions and Discussion

We now revisit our research question: What do undergraduate and graduate science students report on their well-being and psychological need support during predominantly online education? We have attempted to answer this question by analyzing three different sets of data: quantitative data from an online questionnaire and qualitative data from online open ended questions and a student focus group.

### 5.1 Lack of structure, loneliness, and worry

The quantitative analysis of the Likert-scale items shows that students report a clear loss in social contact and difficulties with online education in terms of concentration and structuring their working day. They also worry more but their worries are mainly directed at family, friends, and their course of study rather than on personal health.

Using factor analysis we extracted four main components from the answers: *problems with studying*, *personal worries*, *personal well-being*, and *societal worries*, the latter meaning worries about the societal future. The first of these, problems with studying, can be seen as mainly related the basic psychological need of competence. Online education can contribute, as we hypothesized, to a greater autonomy and this is reflected in the answers to open ended question a (see below). However, in the answers to the Likert-scale items we can see that the greater autonomy is negated by a perceived lack of competence, as students do not report effective studying behavior and difficulty in planning and concentrating. They apparently find it difficult to deal with the offered autonomy. The factor personal well-being relates strongly to the basic psychological need of relatedness. Students report increased feelings of loneliness and depression having a negative impact on their overall feeling of well-being. On the basis of the Likert-scale items, we can therefore conclude that students are thwarted in their need for competence and relatedness. In the end students reported an overall decrease in well-being, reflected in loneliness, helplessness, worrying, and problems with structuring their working days. These issues are elucidated in the answers to the open ended questions.

### 5.2 Autonomy supported, competence and relatedness thwarted

The results of the bottom-up coding of the answers to the two open ended questions in the questionnaire (perceived advantages and suggestions for improvement) paint a clear picture, especially when the categories are binned into larger categories that correspond to the basic psychological needs of autonomy, competence, and relatedness.

In offering perceived advantages of online education, just under one-half of the quotes is related to autonomy in terms of reduced travel time, a resulting surplus of time as compared to pre-corona conditions, and a resulting perceived increase in flexibility. Education could suddenly be enjoyed at home, in bed, out in nature, and often at a time of the students’ own choosing. A smaller percentage of the quotes (19 %) is related to competence in terms of less distractions and learning about online education. The advantages of all lectures being recorded, as implemented by the science faculty during the pandemic, also falls into this category. Students appreciated the fact that it allowed them to rewind and re-watch (difficult or interesting) parts of the lecture. Relatedness was mentioned in less than 10% of the quotes, indicating a higher quality of relations with a smaller group of people and increased awareness of things that truly matter in life. The remainder of the quotes was related to limiting the spread of infections and a better sleep/wake balance due to social distancing.

In terms of our research question, the answers to the open ended questions indicate that the students' well-being was positively influenced by the perceived autonomy in terms of flexibility in organizing their days. Reduced travel time was perceived as an advantage, since it led to more time for other activities beside study. The recording of every lecture during the lockdown supported the feeling of competence. The advantages in terms of relatedness, albeit small in number, are also interesting, since they refer to aspects that may be easily overlooked, such as the fact that even though the quantity of social interactions decreased, the quality of these interactions was actually sometimes perceived to improve during lockdown. More quality time was spent with less people, and there was much less ‘fear of missing out’.

Students were also explicit in their recommendations. The distribution along the psychological needs is very different from the perceived advantages, however. Students refer to measures related to relatedness in over 40% of the quotes, offering suggestions such as returning to on-campus activities as soon as possible, organizing online social events, and offering one-on-one psychological support. The fact that the quality of some social interactions improved during lockdown apparently did not offset the need for more, offline social interaction. Almost one-half of the suggestions referred to competence, for example -interestingly- in the form of support for *teachers* to help them improve their online education. Apparently, students saw their teachers struggle with transferring their teaching practices to the online realm and realized that they needed to be supported in this area. Furthermore, better and more frequent communication and leniency in terms of regulations and deadlines were asked. The university did send almost weekly ‘corona-updates’, but evidently these were not always adequately translated into information that was directly useable by the students. Furthermore, leniency was asked by the students in view of the unavoidable delays and confusion caused by the sudden transition to fully online education, and the fact that some, practical assignments had simply become impossible. Lastly but importantly, students asked for support in how to organize their study at home,. It appears to be difficult for these science students to organize their days and schedules when the very concrete rhythm of going to campus for lectures, working groups, and study, is absent.

The results of the focus group discussion closely mirror the results of the open ended questions: in positive terms, autonomy was most often mentioned, e.g., being able to plan your own day, work according to your own day/night rhythm, and experience more flexibility in general. A few quotes also reflect improved relatedness in the sense that existing relations deepened during the corona period. Negative comments, however, dominated the focus group discussion.

It appears to be predominantly the lack of structure during a period of online education that is detrimental to perceived competence for students. The absence of a daily routine of actually going to university and having scheduled meetings was described as leading to aimless behavior and procrastination. Quick and effective, physical meetings with supervisors and other staff members were also sorely missed. It was reported to be ‘…much easier for me to just go into [my supervisor's] office and talk face to face and it takes even less time compared to like sending emails or [trying to schedule an online meeting].’ In short, students in the focus group confirmed the impression that a period of online education requires student support in terms of organizing and planning – support in perceived competence. The rhythm and efficacy of physical meetings are sorely missed.

Not surprisingly in view of what we have seen so far, students in the focus group were especially negative on relatedness support. Students mentioned increased loneliness, irritation, alienation, and even depression. As one student eloquently put it: ‘…because it is the small connections that make quite a difference in the general well-being.’ Indeed, according to these students, relatedness cannot be supported by online meetings, however well-intended. It requires the quick and fluid interaction of a physical presence.

Generally we can conclude that under the corona regulations, students saw their well-being deteriorate in several areas. They had problems organizing their days, reported more loneliness and stress, and worried more about their study and close ones. In terms of basic psychological needs, students were predominantly positive on perceived autonomy support by the flexibility offered by online education. However, the psychological needs of competence and relatedness were seriously undermined during times of lockdown and online education, mainly because of poorer interaction, lack of structure, loneliness, helplessness, and even depression. Students indicate that they need more support from the university in these areas, for example by on-campus meetings whenever possible, psychological support, improvements in online education, adequate communication, and leniency in the interpretation of regulations and deadlines.

These conclusions confirm our hypothesis that was constructed on the basis of Basic Psychological Need Theory.

### 5.3 Limitations

This study started with the interpretation and analysis of a questionnaire that had already been administered by the student advisor of the science faculty. It featured a satisfactory response rate (32%) for an online, voluntary questionnaire. Since the questionnaire included open ended questions, however, we were able to use the theoretical lens of self-determination theory as the great majority of quotes could be related *post hoc* to one of the three universal psychological needs in BPNT (Vansteenkiste et al., 2020). Based on this analysis we designed a follow-up focus group interview which was explicitly based on BPNT and was thus coded top-down. Data saturation in this focus group is not to be expected given the small number of participants (Almeida et al., 2017), but it was decided to include the data despite this small number, because: (1) the participants did cover undergraduate, graduate, and international students, (2) the focus group results closely mirrored but enriched the results from the open ended questions, and (3) the interrater reliability was nearly perfect for all second coding.

The students in our sample were all science students in the fields of mathematics, physics, chemistry, information science, and life sciences. As there were no students in other fields, it is possible that the results are biased. Furthermore, apart from the academic level (undergraduate and graduate), no demographical data were collected in the questionnaire. Therefore any possible differences based on gender, age, nationality, etc., could not be studied. This constitutes another possible source of bias. However, as BPNT addresses *universal* human psychological needs (Ryan and Deci, 2017), these factors are not expected to greatly influence the results. In other words, we expect our results to be generalizable to a wider student population. This expectation is reinforced by the fact the other studies have reported similar results on student well-being in times of online education (Arslan and Allen, 2021; Bakker et al., 2021; Ellaway et al., 2020; Ellis et al., 2020; Genç and Arslan, 2021; Meulenbroeks et al., 2021).

### 5.4 Implications

The 2020 pandemic may not be the last one. Indeed, disruptions like this may become part of the new normal (Davis et al., 2021) and constant stress may become the new normal (Hoyt et al., 2021). This study concludes that in order to prevent a sharp deterioration of student well-being during social distancing and online education, measures need to be put into place to support competence and relatedness.

Fortunately, online education with its almost inherent flexibility and adaptability can readily support the basic need for autonomy. However, as experience with Massive Open Online Courses (MOOC's) had shown, support of autonomy in itself is not enough to guarantee a satisfying educational experience (Martin et al., 2018; Onah et al., 2014; Semenova, 2020).

This study offers relatively differentiated suggestions for the support of the other two universal psychological needs. Students’ perceived competence can be supported by offering

synchronous meetings whenever possible, online if need be, and by recording these meetings. Interaction needs to be made as direct and diverse as possible within the limits of online platforms, including the use of breakout rooms and feedback tools. In organizing this high-quality online education, however, teachers need support in terms of webinars, and professional development communities. Students need support in their study skills: how to structure your day when there is no explicit rhythm? How to organize your work-from-home environment? Students further ask for timely, concise, and correct communication on all matters concerning their study as well as empathy for their situation and a corresponding leniency in applying regulations, deadlines, and procedures.

Students' sense of relatedness came out as a very important factor in students’ well-being. According to the students in the sample, universities have a responsibility in providing socio-emotional support for their students as well as support on the academic level. Even though students generally agree that there is no substitute for the high quality of offline interaction, in times of lockdown they would welcome online social gatherings with their peers. In dealing with increased stress levels due to social distancing they require one-on-one psychological support, online if need be. In the end, however, our results warrant the overall conclusion that universities should strive to provide as much offline meetings as possible within the regulations.

Our results relate to the extreme situation of a lockdown because of the corona pandemic. However, also in less extreme situations online education is on the rise. Results of the current study may also apply to general situations in online education, in which competence and relatedness and their support may come less natural than during on-campus education.

### 5.5 Future research

The results of this study confirm earlier observations that social distancing and online education during the pandemic lead to stress and reduced well-being and motivation for study (e.g., Eringfeld, 2021; König et al., 2020; van Schalkwyk, 2021). The above suggestions for improving well-being by supporting universal psychological needs need to be carefully translated into interventions that subsequently need to be evaluated. New research questions proposed are: How can teacher support be organized in ways that does not add to the already notoriously heavy teacher work load? How can one-on-one psychological support for students be effectively organized, online or offline? What are the effects of online social events during a prolonged lockdown in terms of basic psychological need support? How can students be supported in organizing their daily schedules without physical on-campus meetings indicating the rhythm?

Finally, as our sample was substantial but consisted entirely of science students, this study would warrant replication with a more diverse student population. Further research into possible differences related to gender or social economic status (SES) could also be considered.

## 6 Ethical statement

This study complies with the regulations of the ethical board of the Faculty of Science and Geology of Utrecht University. The authors explicitly deny any conflict of interest in the context of this study.

## 7 Interview protocol

**Figure 6:**
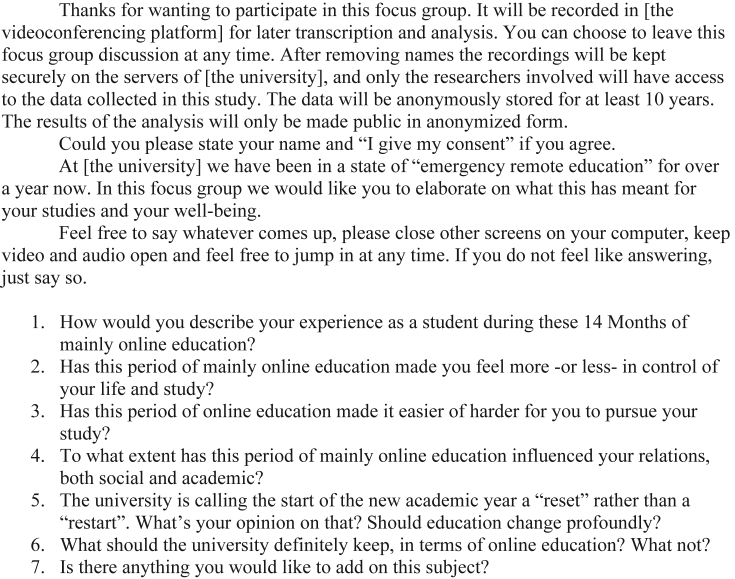
The full interview protocol as used during the focus group interview on student well-being.

The full interview protocol is given in Figure 6 below.

## Declarations

### Author contribution statement

Ralph Meulenbroeks: Conceived and designed the experiments; Performed the experiments; Analyzed and interpreted the data; Wrote the paper.

Wouter R. van Joolingen: Conceived and designed the experiments; Analyzed and interpreted the data; Wrote the paper.

### Funding statement

This research did not receive any specific grant from funding agencies in the public, commercial, or not-for-profit sectors.

### Data availability statement

Data will be made available on request.

### Declaration of interests statement

The authors declare no conflict of interest.

### Additional information

No additional information is available for this paper.
